# 3D printed testing aids for radiographic quality control

**DOI:** 10.1002/acm2.12574

**Published:** 2019-04-06

**Authors:** Kent M. Ogden, Kristin E. Morabito, Paul K. Depew

**Affiliations:** ^1^ Department of Radiology SUNY Upstate Medical University Syracuse NY USA

**Keywords:** 3D printing, additive manufacturing, fluoroscopy, phantoms, quality control, radiography

## Abstract

Quality control testing of radiographic and fluoroscopic imaging systems requires positioning of test objects in the x‐ray beam in a precise and repeatable fashion. In this work we present several three‐dimensional (3D) printed testing aids that improve efficiency, accuracy, and repeatability of quality control testing. We also present a new device for determining the location of the perpendicular ray in radiographic systems. These devices were designed in an open source software program (OpenScad, http://www.openscad.org) and 3D models were saved in .stl format for printing. The models were printed on either a MakerBot Replicator 2 or Replicator Z18 printer (MakerBot Industries, LLC, Brooklyn, NY). The testing aids were printed using polylactic acid (PLA) filament. To investigate the radiographic characteristics of the PLA used, test articles were printed and used to measure the half‐value layer (HVL) thicknesses in mm of PLA and half‐value densities (HVD) in g/cm^2^ of PLA for two different colors and over a wide range of radiographic beam qualities, using a portable fluoroscopic c‐arm system. HVL thicknesses of clear PLA ranged from approximately 20 mm at 50 kV nominal tube voltage to 27 mm at 120 kV nominal tube voltage. For green PLA, the HVL thickness was 19 mm at 50 kV tube voltage and 25.7 mm at 120 kV tube voltage. The HVD of clear PLA ranged from 2.37 g/cm^2^ at 50 kV nominal tube voltage to 3.19 g/cm^2^ at 120 kV nominal tube voltage. For green PLA, the HVD was 2.35 g/cm^2^ at 50 kV tube voltage and 3.17 g/cm^2^ at 120 kV tube voltage. The cost of the devices range from under $2 to approximately $20 in materials. The files used to create the models are freely available at https://github.com/Upstate3DLab/3D-Printed-Radiographic-Test-Tools.

## 1 INTRODUCTION

Quality control (QC) testing of radiographic and fluoroscopic equipment is an important part of quality assurance (QA) programs, ensuring that the imaging equipment is functioning properly and providing maximum benefit to the patient relative to the radiation dose delivered.^1^ State regulations generally require QA programs that include equipment performance validation. Federal regulations concerning the capabilities of x‐ray equipment, especially fluoroscopic systems, also play an important role in the specific tests that are performed regularly on imaging equipment.^2^

Quality control (QC) testing may be categorized into five general areas: (a) Mechanical inspection of the equipment; (b) Beam geometry tests such as light field, collimator, and central ray alignment; (c) Beam quality, tube output, and patient exposure tests; (d) Systems tests such as automatic exposure control, exposure index, and timer accuracy; and (e) Image quality tests such as detector linearity, resolution, and low contrast detectability. Not all of these tests are independent of each other, for example patient exposures depend on beam quality, tube output, as well as automatic exposure or automatic brightness control performance.

Some of these tests require specialized equipment, which may be difficult or impossible for an individual to manufacture or improvise, such as precision spatial resolution test targets. Several companies offer specialized equipment to meet the needs of radiographic testing, but there are tests that could benefit from positioning aids or other devices for which there are few commercial products available.

Additive manufacturing, more commonly referred to as three‐dimensional (3D) printing, provides a low cost, readily available technology that allows Medical Physicists to rapidly prototype and produce custom devices to meet specific testing needs. There has been significant growth in the use of 3D printing for creating models of patient anatomy for surgical planning, for creating anthropomorphic phantoms, and for creating QC tools in radiation therapy.^3^, ^4^, ^5^, ^6^, ^7^, ^8^ However, to date there has been little reference in the literature to the use of 3D printing to create aids for radiographic and fluoroscopic quality control testing.^9^

Three‐dimensional (3D) models may be derived from scans of existing objects or designed from scratch using 3D modeling software. For scanned objects, the scan data usually requires segmentation, then model generation, which creates a surface mesh representation of the segmented region. This functionality is becoming increasingly available in commercial 3D post‐processing platforms used in Radiology departments, and there are open source programs with this functionality, such as 3D Slicer.^10^ There are also open source programs available, such as Blender^11^ and OpenScad,^12^ that allow users to digitally design 3D models. Surface models are processed by the 3D printer's slicing software to produce a print file that is used to print the physical object. Commonly available 3D printers use a Fused Deposition Modeling (FDM) process that involves plastic filaments such as polylactic acid (PLA) or acrylonitrile butadiene styrene (ABS). These materials are suitable for making reasonably durable objects at a low cost.

Radiographic QC testing often requires positioning test objects accurately and securely in the x‐ray beam. An example is the positioning of aluminum and copper phantoms used to estimate patient exposure during fluoroscopic procedures. Tests may require specific spacing from image receptor or x‐ray source, and x‐ray dosimetry sensors must be positioned precisely and securely to ensure accurate and repeatable results. Testing of different equipment types, such as portable C‐arm fluoroscopes, fixed interventional units, or radiographic/fluoroscopic (R/F) rooms also presents different needs for positioning phantoms and measuring equipment. 3D printing technology allows us to address the various requirements for testing these differing equipment types.

In this work we present several 3D printed devices to aid in QC testing of radiographic and fluoroscopic equipment. We have created tools that aid in collimation testing and for general positioning of test articles such as aluminum blocks used for dosimetric measurements and commercial radiographic and fluoroscopic image quality phantoms. Collimation test tools include holders for radiochromic filmstrips that allow for easy positioning on fluoroscopic image receptors, and a newly designed tool to measure the x‐ray perpendicular ray relative to the center of a radiographic image receptor or x‐ray field central ray.

Any devices used as QC aids should ideally have minimal impact on the automatic exposure control or automatic brightness control systems. This is primarily achieved by designing devices that introduce a minimal amount of material into the beam, and by using materials that have low attenuation. PLA is an organic compound (C_3_H_4_O_2_)_n_ and therefore has mass attenuation properties similar to tissue. To better understand the characteristics of PLA being used in our testing aids, we have measured the material attenuation characteristics over a range of beam energies, and we present that data here for comparison with previously published work.^13^, ^14^

## 2 MATERIALS AND METHODS

### 2.A Quality control testing aids

Three‐dimensional (3D) printed objects are made from digital files that represent the surface of the model. The most commonly used file format for 3D models is the .stl format (stereolithography or Standard Tessellation Language). For our work, the models were originally designed using Blender version 2.79 (http://www.blender.org), and the finalized designs were modeled in OpenScad version 2015.3‐02 (http://www.openscad.org). One component of our models, used to hold the x‐ray detector for our dosimetry equipment, was initially created by scanning the vendor‐supplied holder in a computed tomography (CT) scanner and generating a surface model of the holder from the CT scan. This surface model was modified to create a removable probe holder that could be attached to the testing aids. A *de novo* version of the probe holder model was also created in OpenScad using the dimensions of the vendor‐supplied holder.

Models were printed on either a MakerBot Replicator 2 or MakerBot Replicator Z18 printer (MakerBot, Brooklyn, NY). All models were printed using PLA filament using a 0.2 mm layer thickness and infill density of 15%. All models and attenuation test article print files were generated using MakerBot Desktop version 3 software. The models were oriented such that the minimal amount of support material was needed and to prevent use of support material in locations that would be difficult to clean after printing was complete, and to conserve filament. The models were generally designed to minimize or eliminate the presence of PLA in the direct x‐ray beam, however, it is not possible to do this in all cases. A good example of this is that the holders designed for the dosimetry system detector must be in the direct beam when being used.

### 2.B PLA attenuation measurements

To measure the attenuation of PLA, 5 × 5 × 0.5 cm test articles were printed at 100% infill density using the MakerBot Replicator 2 printer. The extruder temperature was set to 230°C with all extruder speeds set to 50 mm/s. Layer height was set to 0.2 mm with a linear infill pattern. Rafts were used to provide a uniformly flat surface to build the test articles on, and no supports were needed.

Polylactic acid (PLA) filament with a nominal 1.75 mm diameter was purchased from two separate vendors. The green PLA was manufactured by Maker Shed (Make: San Francisco, CA), and the natural filament was manufactured by 3D Solutech (http://www.3dsolutech.com). Filament diameter was measured in eight separate locations using a micrometer caliper for each of the filaments. The test article lengths, widths, and thicknesses were measured using a Vernier caliper. The lateral dimensions of the test articles were used to calculate their areas in cm^2^. The test article masses were measured using a Sartorius Type 2255S0400 digital scale. The masses and areas were used to calculate the areal density of the individual articles.

**Figure 1:**
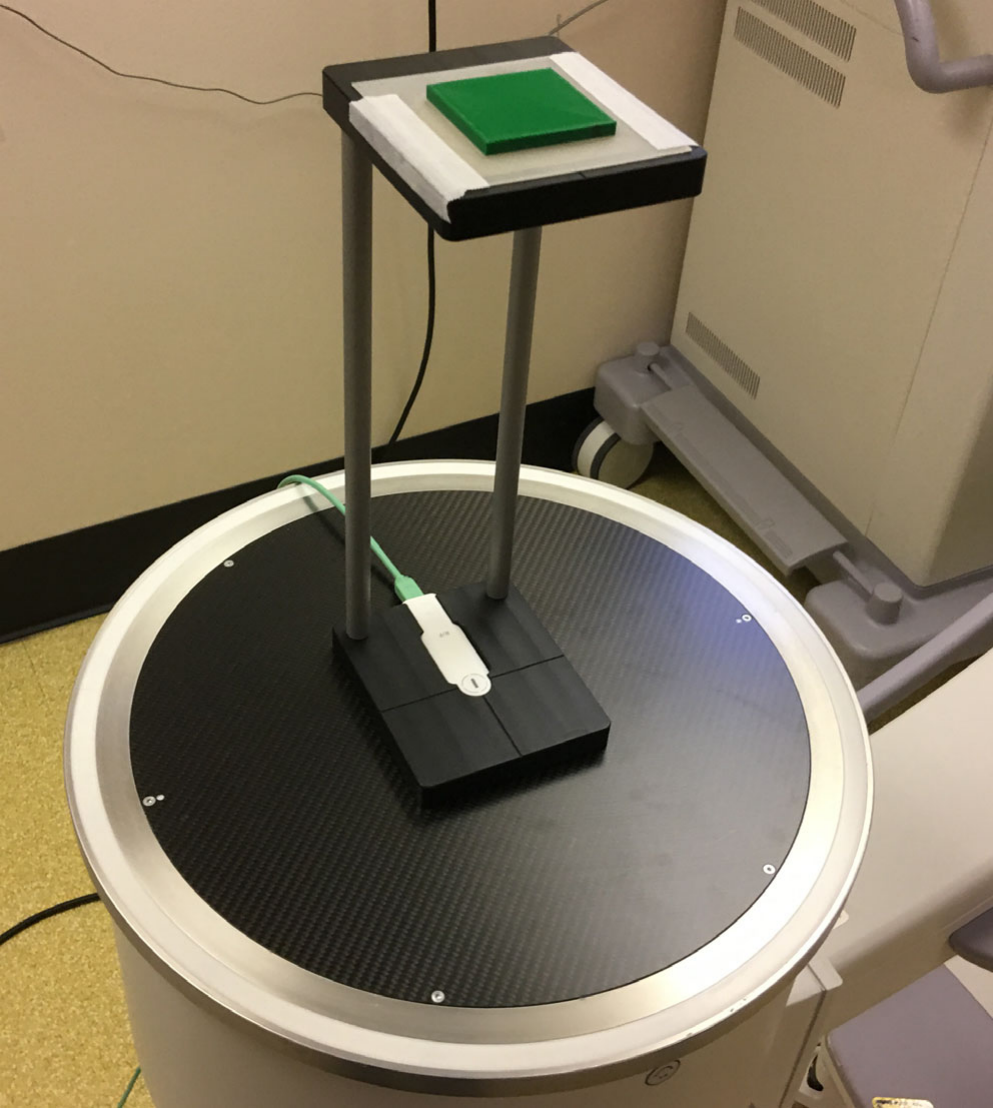
Polylactic acid test article setup for measuring half‐value layer.

X‐ray transmission was measured using an OEC 9900 portable fluoroscopy unit (GE Medical Systems, Waukesha, WI). Exposure rates in mGy/min were recorded using a RaySafe X2 dosimetry system with the R/F sensor (Fluke Biomedical, Cleveland, OH). The stand provided by the manufacturer for making HVL measurements was used as shown in Fig. 1. The opening on the top platform of the HVL stand was larger than the size of the test articles, therefore a thin platform with a 4 cm square opening was 3D printed and taped to the top of the HVL test stand. The PLA test articles were centered on the opening as shown in Fig. 1. The distance from the bottom of the PLA test article to the detector is about 30 cm. The x‐ray beam was collimated to be just larger than the active area of the x‐ray detector to maintain a narrow‐beam geometry.

Air kerma rate values were recorded for three separate exposures for PLA nominal thicknesses of 0 to 5 cm in 0.5 cm increments. Measurements were made for nominal tube voltages from 50 to 120 kV in 10 kV increments. The beam HVL thicknesses as reported by the RaySafe system in millimeters of aluminum were recorded at each of the tube potentials with no PLA in the beam. Half‐value layer thicknesses of PLA were calculated for all beam qualities using a second order exponential fit to the average air kerma rate vs PLA thickness data using SigmaPlot 11 (Systat Software, Inc., San Jose, CA). The areal density of PLA required for 50% attenuation was also calculated and expressed in units of g/cm^2^ using a second order exponential fit. We refer to this value as the Half‐Value Density (HVD). All measurements and calculations were repeated for both clear (natural or uncolored) and green PLA.

To explore the effect of the positioning aids on technique, measurements were made using a Siemens Axiom Artis interventional radiology (IR) unit with and without the 3D printed IR phantom holder, and with and without the dosimetry detector holder mounted on the IR phantom holder. This device has the most material positioned in the beam during its use.

## 3 RESULTS

### 3.A Collimation test tools

#### 3.A.1 Filmstrip holder

**Figure 2:**
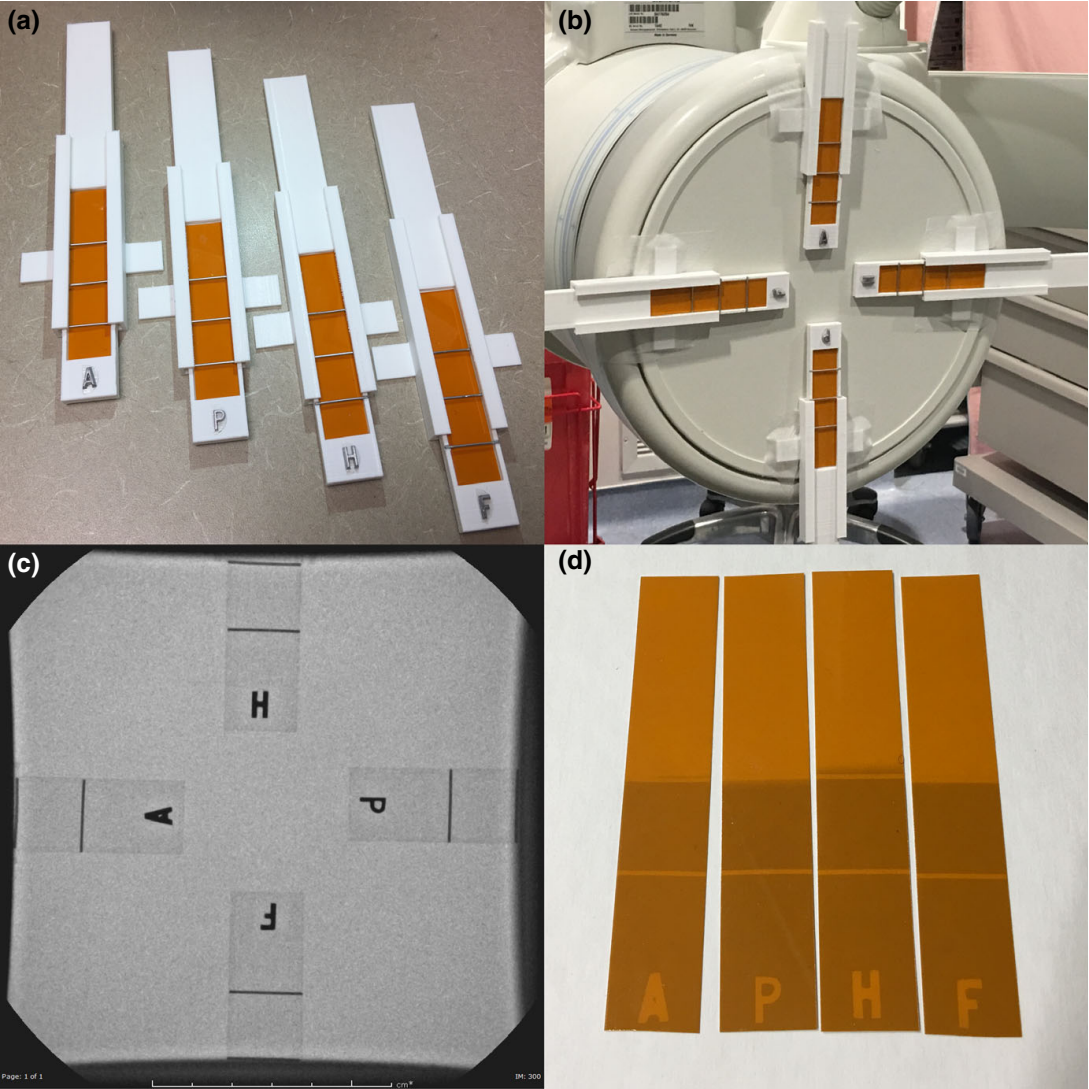
(a) Radiochromic filmstrip holder sets, (b) a typical use case in an interventional room, (c) aligned holders shown fluoroscopically, and (d) the resulting exposed film. Note that the wires in this example were positioned roughly at the edge of the collimator and not at the edge of the image receptor so that they would be visible in the fluoro image.

A filmstrip holder was designed to hold radiochromic filmstrips for determining x‐ray field position in radiographic or fluoroscopic equipment. Pictures illustrating the device and its use are shown in Fig. 2.

The filmstrip holder consists of two parts: A track that is mounted to the image receptor using tape or other adhesive, and the actual film holder that slides in the track for positioning. There are strips of loop‐type material used in hook and loop fasteners on the inside edges of the track that provide enough friction to prevent the film holder from sliding out. The filmstrip holder has three fiducial wires made from paper clip wire at 2 cm intervals, as well as lead letters that will be visible on the fluoroscopy monitor and the exposed film.

During use, the film holder is positioned under fluoroscopy such that one of the wire markers is at the edge of the image field. The exposure is continued using cine or digital subtraction angiography mode until the film is sufficiently darkened to indicate the location of the x‐ray field edge. The field size may be measured while the filmstrip holders are still in the tracks, and the x‐ray field to image receptor alignment is measured after removing the film from the holders.

#### 3.A.2 Perpendicularity test tool

**Figure 3:**
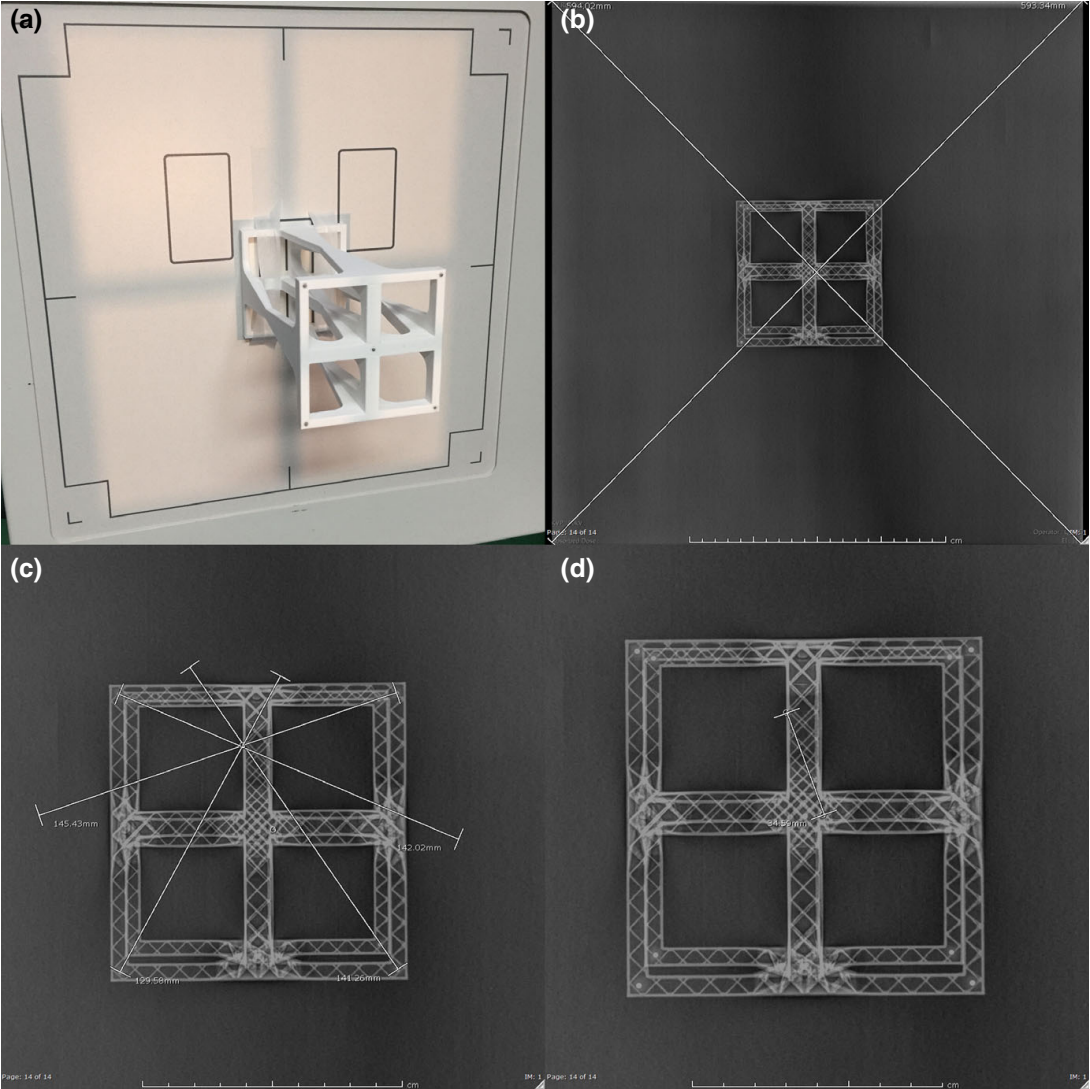
Perpendicularity test tool. Images show (a) the test tool taped to an upright image receptor, (b) the resulting radiograph acquired with a flat panel detector with diagonal lines indicating the center of the image receptor, (c) the fiducial markers connected by lines drawn on a PACS workstation showing the location of the perpendicular ray, and (d) a final measurement of the distance between the perpendicular ray and the detector center. The intersections were marked with letters “O” and “C” using the annotation tool on the PACS workstation.

We have developed a perpendicularity test tool to allow accurate determination of the x‐ray perpendicular ray position on radiographic image receptors. The tool consists of four pairs of radiopaque markers (1.5 mm mammography nipple markers) aligned vertically at the top and bottom corners of a 10 cm square, 20 cm tall platform. There is also a single fiducial marker at the center of the top platform. Images showing the device and typical use are shown in Fig. 3.

To use the test tool, it is positioned at the center of the light field by aligning the light field crosshair with the central fiducial marker on the tool. A radiograph is acquired using a low tube potential (~60 kV or lower) and an exposure technique of ~2 mAs. The projections of the vertically aligned fiducial markers lie on lines that run through the perpendicular ray (the location in the image where the x‐ray beam is perpendicular to the image receptor). A minimum of two sets of markers are required to locate the perpendicular ray. Precision is improved by using four sets of markers and estimating the position of the perpendicular ray as the center of the overlap points created by drawing lines through the four pairs of markers as shown in Fig. 3.

The perpendicularity tool provides measurements of multiple quantities. The central fiducial marks the center of the light field, and can be used to measure the deviation of the light field center to the center of the detector or center of the x‐ray field (the central ray location). The perpendicular ray distance to the central ray or detector center may be measured, as well as the distance between the light field center and the perpendicular ray location.

### 3.B Phantom positioning aids

**Figure 4:**
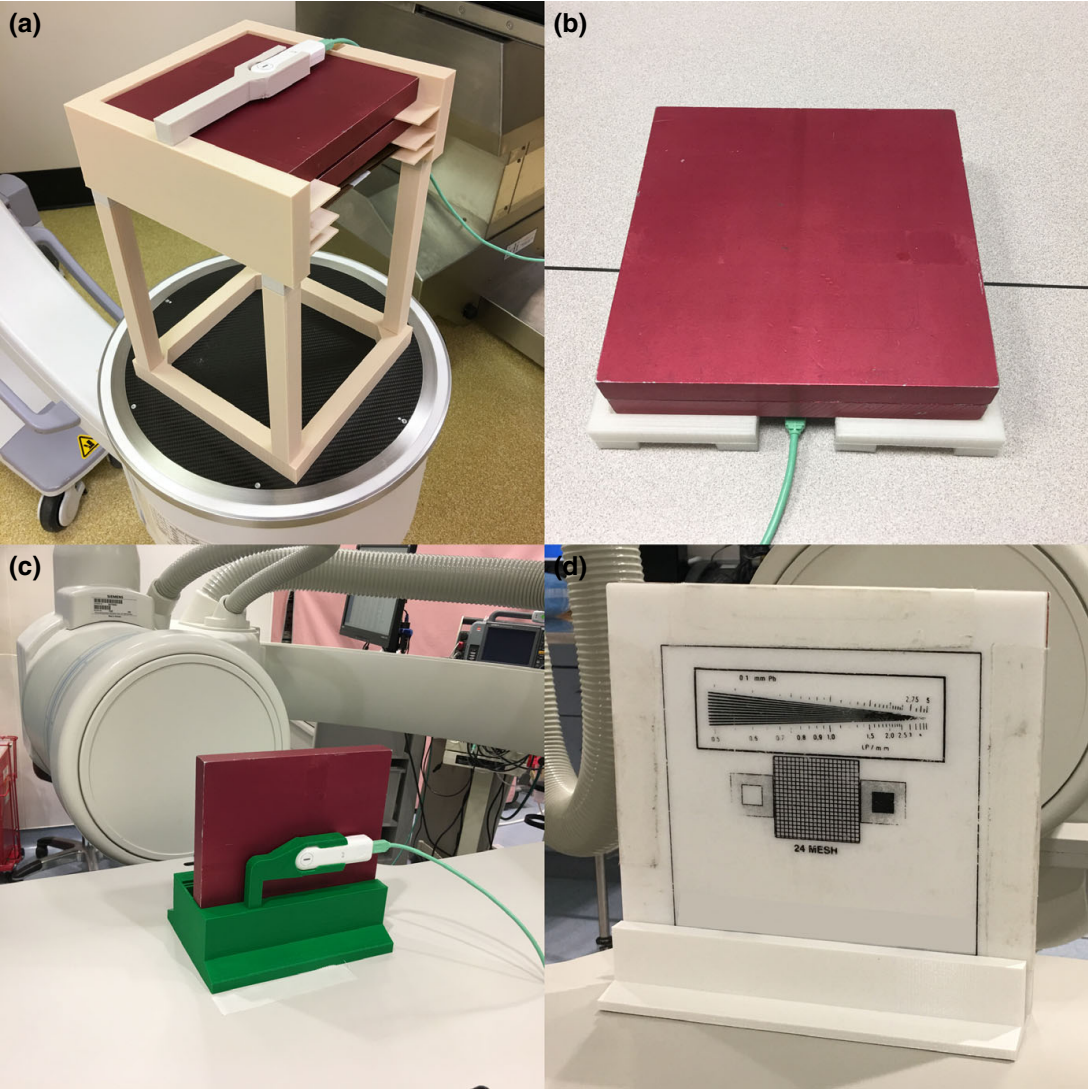
Positioning aids for (a) portable c‐arm fluoroscopes, (b) R/F rooms with under‐table x‐ray tube, (c) interventional c‐arms in the lateral position, and (d) an image quality phantom holder for use in fluoroscopy or radiography.

Positioning aids were designed to hold standard test objects used in fluoroscopy and radiography. Standard objects include aluminum blocks with dimensions 18 × 18 × 19 mm, copper sheets of 0.5 and 1 mm nominal thickness, and lead beam blockers of nominal 5 mm thickness. Images of the positioning aids in use are shown in Fig. 4.

The positioning devices (except for the image quality phantom holders) have an x‐ray detector holder. For the IR and c‐arm phantom holders, the x‐ray detector holder may be removed if desired. This detector holder is specific for our equipment, but it would not be difficult to design additional holders for detectors from other manufacturers.

### 3.C Electrometer stand

**Figure 5:**
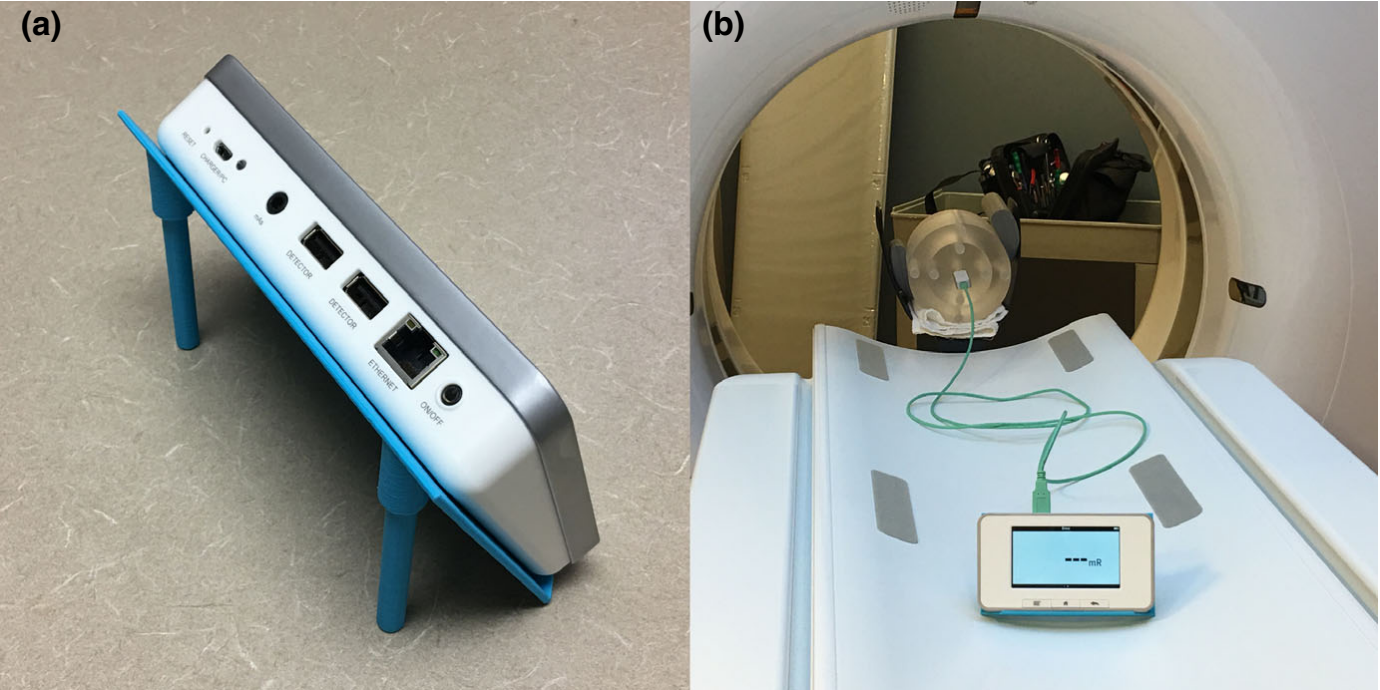
(a) Dosimetry base unit stand, (b) base stand being used in a computed tomography scanner.

The last device we designed was a stand for the base unit of our dosimetry system. This allows for convenient positioning of the base unit to improve viewing of the display from a distance. This has proven to be especially useful in radiographic room and CT testing. The device is shown in Fig. 5.

### 3.D PLA attenuation results

Polylactic acid (PLA) filament diameters (mean ± 1 standard deviation) were 1.73 ± 0.011 mm for the green PLA and 1.76 ± 0.016 mm for the natural PLA. These diameter differences are statistically significant, with a *P*‐value of 0.003. The mean areas for the natural and green PLA articles were 25.2 ± 0.23 cm^2^ and 25.1 ± 0.18 cm^2^, respectively. The measured mass for each article was used to calculate the article's areal density. The areal densities for the natural and green PLA articles were 0.590 ± 0.0047 g/cm^2^ and 0.614 ± 0.0044 g/cm^2^, respectively (*P* < 0.001). The measured thicknesses of the test articles were about 1% greater for the green PLA, however, the areal density was about 4% greater for the green PLA test articles.

**Figure 6:**
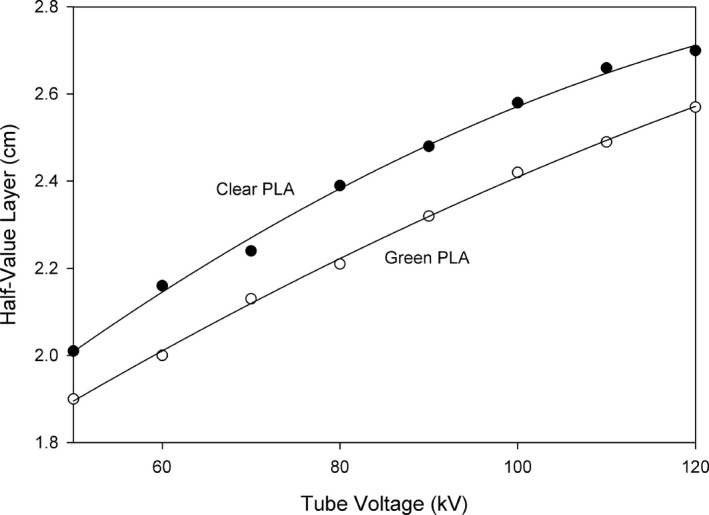
Half‐value layer thickness of polylactic acid (PLA) vs nominal tube voltage for clear and green PLA.

**Figure 7:**
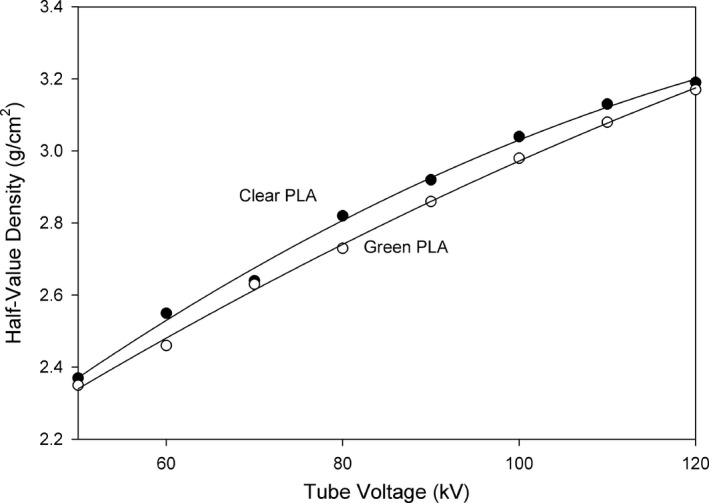
Polylactic acid (PLA) half‐value density as a function of nominal tube voltage.

The PLA half‐value layer (HVL) thicknesses were solved for using a second order exponential equation fit to the measured transmission data. Half‐value layer thicknesses were calculated using the nominal PLA thickness in cm. The PLA half‐value densities were calculated in g/cm^2^. Plots of the HVL thicknesses are shown in Fig. 6, and the half‐value density plots are shown in Fig. 7. The curves in Figs. 6 and 7 were fit with the second order equation $HVL\;or\;HVD=a\;kV^{2}+b\;kV+c$. The parameters for the curve fits are shown in Table 1.

**Table 1:** Curve‐fit parameters for half‐value layer (HVL) and half‐value density (HVD) as a function of tube voltage

Material	a	b	c
Clear PLA HVL (cm)	−6.07 × 10^‐5^	2.04 × 10^‐2^	1.14
Clear PLA HVD (g/cm^2^)	−6.79 × 10^‐5^	2.34 × 10^‐2^	1.37
Green PLA HVL (cm)	−3.10 × 10^‐5^	1.49 × 10^‐2^	1.23
Green PLA HVD(g/cm^2^)	−3.57 × 10^‐5^	1.80 × 10^‐2^	1.53

PLA: polylactic acid.

**Figure 8:**
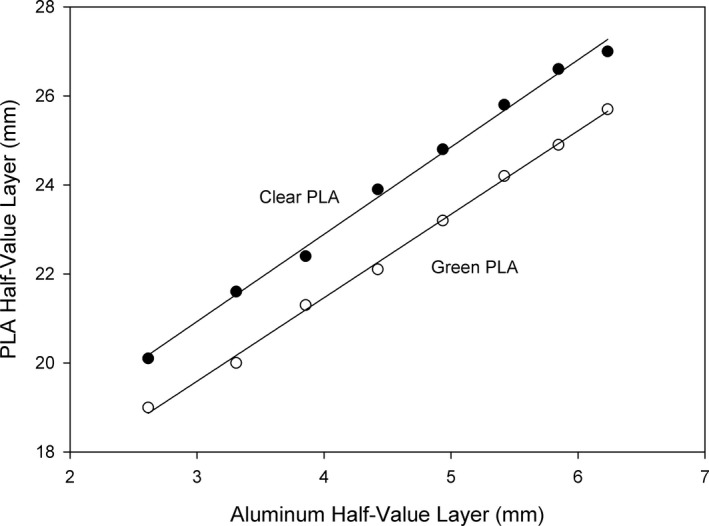
Best‐fit straight lines to the polylactic acid (PLA) half‐value layer (HVL) vs the Al HVL.

To compare the PLA HVL to the reported HVL in millimeters of aluminum, the PLA HVL thicknesses were plotted against the aluminum HVL thicknesses, as shown in Fig. 8. The equations of the best fit lines are HVL_PLA_(mm) = 1.96 × HVL_Al_ + 15.1 for the clear PLA, and HVL_PLA_(mm) = 1.88 × HVL_Al_ + 14.0 for the green PLA.

Using the measured HVL thicknesses of clear PLA and the HVL aluminum thicknesses reported by the dosimetry system, the linear attenuation coefficients, *μ*, were calculated as $\mu =\frac{\ln(0.5)}{t_{\frac{1}{2}}}$. The calculated values of *μ* are listed in Table 2.

**Table 2:** Half‐value layer (HVL) values for polylactic acid (PLA) and Aluminum, and the calculated attenuation coefficients at each kV

kV	Clear PLA HVL (mm)	Clear PLA μ (mm^−1^)	Aluminum HVL (mm)	Aluminum μ (mm^−1^)
50	20.1	0.0345	2.6	0.267
60	21.6	0.0321	3.3	0.210
70	22.4	0.0309	3.9	0.178
80	23.9	0.0290	4.4	0.158
90	24.8	0.0279	4.9	0.141
100	25.8	0.0269	5.4	0.128
110	26.6	0.0261	5.8	0.119
120	27.0	0.0257	6.2	0.112

## 4 DISCUSSION

The 3D printed positioning aids presented here are lightweight, easily transported, and have improved our QC testing process. Prior to the development of these tools, we had used improvised positioning aids such as cardboard boxes, blocks of foam, etc. These improvised devices were not very stable, and it was time‐consuming to position test articles and dosimetry sensors at a precise distance from the image receptor and with the dosimetry sensor centered on the phantoms. Differences in room and image receptor designs also created positioning problems that our custom devices address. We have designed dedicated positioning devices for upright image receptors, which had been particularly difficult to deal with. For most of our QC aids, the x‐ray sensor holder is removable when not needed. This is useful when performing some image quality tests, such as with a low contrast target used in conjunction with the attenuation plates. The 3D printed testing aids are also an improvement with respect to infection control issues, as they are wipeable with disinfecting wipes. There is no way to disinfect the porous surfaces of cardboard or foam devices to hospital standards.

The perpendicularity test tool represents a potential improvement over other common methods of evaluating beam geometry relative to the image receptor. This tool allows for the measurement of the deviation between the x‐ray perpendicular ray and the central ray or detector center, and the deviation between the center of the light field and x‐ray perpendicular ray, image receptor, or central ray. We are currently investigating the utility of these measurements vs traditional beam geometry measurements.

One drawback of commercially available tools for measuring the x‐ray central ray perpendicularity is that they are designed to be used at a specific source‐image distance (SID), typically 100 cm. They are also positioned by using the light field center, which may not coincide with the central ray, and the results may be a simple pass/fail instead of the quantitative results obtained using our test device. By using multiple sets of aligned fiducial markers, our tool allows us to determine the position of the perpendicular ray regardless of the SID. Our tool is easily used on an upright image receptor, which is difficult to do with commercial test tools.

The radiochromic filmstrip holders allow for easy positioning of the wire markers either relative to a light field or to the edge of the image receptor when used in fluoroscopy. Because of their design, it is easy to move the holder during fluoroscopy to get immediate feedback on the position. We find it is difficult with some systems to generate a beam of significant intensity to develop the film in a reasonable amount of time. We have used an attenuator in the center of the image receptor to help drive the technique up, which results in reduced total exposure time.

We have found that the probe holder has a small but measurable impact on the measured air kerma, particularly for the interventional c‐arm positioning aid. We have chosen to ignore this increase as it results in a more conservative estimate of patient exposure, and since the measured exposures are still far below any regulatory limit. It may be possible to reduce this effect by redesigning the probe holder such that the x‐ray sensor is held by its base instead of at the measurement end of the sensor.

Polylactic acid (PLA) is a bio‐derived material and therefore will have a low average atomic number. Our measured HVL thicknesses for two different colors of PLA from two manufacturers show that there are in fact measurable differences in their linear and areal density attenuation characteristics. The linear attenuation differences as reflected by the HVL thickness may be due to physical density differences, printed infill density differences, or average atomic number differences. Since the filament diameter is larger for the natural PLA, it is likely that the printed infill density will be higher than for the green PLA, since the diameter differences were not corrected for in the printer software. The default printer setting of 1.77 mm diameter was used for both filaments. If this resulted in a difference in infill density, then the natural PLA should have a higher printed density and resulting decrease in HVL thickness. The results show that the natural PLA has a larger HVL thickness, indicating that either the physical density, the average atomic number, or both, are lower for the natural PLA.

The areal density differences of the two sets of test articles are not explained by the small differences in the measured thicknesses of the articles, reflecting a difference in physical density. However, since there are differences in the Half‐Value Densities for the two filaments, a possible explanation is that the average atomic number of the filaments is different, since HVD removes the effect of physical density. It is possible that there are other differences in the manufacturing of the two PLA samples that are responsible for these differences. The main point is that differences in attenuation of PLA exist and should be considered when manufacturing items that will be used in an x‐ray beam.

Three‐dimensional (3D) printed models made from PLA are inexpensive. PLA can be purchased for approximately $20 for a 1 kg spool. All of the devices that we present here were made using a total of two spools of PLA. The most expensive model was the portable c‐arm stand which required about one full spool. The least expensive was the electrometer stand, which required less than $1.50 in materials. The c‐arm phantom stand is large enough that it is not printable on many smaller printers. We have therefore designed it in pieces to allow printing on many common printers. The 3D printers used in this work cost approximately $2000 and $6000. This represents a significant investment for an individual, but 3D printers are increasingly available in public libraries and maker spaces.^15^, ^16^, ^17^

## 5 CONCLUSION

Additive manufacturing is a disruptive technology that has had a large and increasing impact in many domains, including healthcare. Medical Physicists can benefit from this technology in multiple ways, such as the manufacturing of custom QC phantoms, patient specific phantoms for dosimetric purposes, and for prototyping novel equipment‐testing devices.

In this work we have presented several new devices that can aid the Medical Physicist or Medical Physicist Assistant in performing routine quality control testing of radiographic and fluoroscopic imaging systems. We have made these models available for download at https://github.com/Upstate3DLab/3D-Printed-Radiographic-Test-Tools. We have posted the OpenScad code and the generated digital models in .stl format. Users may modify the code to customize the devices to address varying phantom dimensions and to accommodate differences in printer characteristics.

## CONFLICT OF INTEREST

The authors declare no conflict of interest.
